# Expression Quantitative Trait Loci (eQTL) mapping for callose synthases in intergeneric hybrids of *Citrus* challenged with the bacteria *Candidatus* Liberibacter asiaticus

**DOI:** 10.1590/1678-4685-GMB-2019-0133

**Published:** 2020-06-15

**Authors:** Maiara Curtolo, Laís Moreira Granato, Tatiany Aparecida Teixeira Soratto, Maisa Curtolo, Rodrigo Gazaffi, Marco Aurélio Takita, Mariângela Cristofani-Yaly, Marcos Antonio Machado

**Affiliations:** 1 Instituto Agronômico de Campinas, Centro APTA Citros Sylvio Moreira, Cordeirópolis, SP, Brazil; 2 Universidade Estadual de Campinas, Programa de Pós-Graduação em Genética e Biologia Molecular, Campinas, SP, Brazil.; 3 Universidade de São Paulo, Escola Superior de Agricultura Luiz de Queiroz, Programa de Pós-Graduação em Genética e Melhoramento de Plantas, Piracicaba, SP, Brazil.; 4 Universidade Federal de São Carlos, Centro de Ciências Agrárias, Departamento de Biotecnologia e Produção Vegetal e Animal, Araras, SP, Brazil.

**Keywords:** Gene expression, molecular markers, polymorphism

## Abstract

Citrus plants have been extremely affected by Huanglongbing (HLB) worldwide, causing economic losses. HLB disease causes disorders in citrus plants, leading to callose deposition in the phloem vessel sieve plates. Callose is synthesized by callose synthases, which are encoded by 12 genes (*calS1*– *calS12*)in *Arabidopsis thaliana*. We evaluated the expression of eight callose synthase genes from Citrus in hybrids between *Citrus sunki* and *Poncirus trifoliata* infected with HLB. The objective of this work was to identify possible tolerance loci combining the expression quantitative trait loci (eQTL) of different callose synthases and genetic Single-Nucleotide Polymorphism (SNP) maps of *C. sunki* and *P. trifoliata*. The expression data from all *CscalS* ranged widely among the hybrids. Furthermore, the data allowed the detection of 18 eQTL in the *C. sunki* map and 34 eQTL in the *P. trifoliata* map. In both maps, some eQTL for different *CscalS* were overlapped; thus, a single region could be associated with the regulation of more than one *CscalS*. The regions identified in this work can be interesting targets for future studies of *Citrus* breeding programs to manipulate callose synthesis during HLB infection.

## Introduction

The citrus industry plays an important role in the productivity chain in Brazilian agribusiness. Brazil is the largest sweet orange producer, and, during the period 2017/18, its yield was approximately 397 million of boxes of 40.8 kg each (Fundecitrus, 2018). Nevertheless, this important economic area has been challenged by Huanglongbing (HLB) (Colleta-Filho *et al*., 2004), which has caused great economic losses because of the fast dissemination and severity. In 2008, 0.61% of the crop trees were symptomatic; in 2016, this number increased to 16.92%. In four years of evaluation, 50% of the scion trees showed disease symptoms, with an approximately 60% decrease in production (Fundecitrus, 2018).

HLB is caused by the gram-negative bacterium *Candidatus* Liberibacter asiaticus (*C*Las) (Colleta-Filho *et al*., 2004), which is restricted to the phloem sieve tubes (Jagoueix *et al*., 1994), and is transmitted by the vector citrus psyllid (*Diaphorina citri*) (Gottwald, 2010). Citrus plants recognize pathogen-associated molecular patterns (PAMPs) of *C*Las, triggering callose deposition in the phloem sieve plates (Gómez-Gómez *et al*., 1999; Luna *et al*., 2011). The deposition of high amounts of callose and phloem proteins (PP2) on the phloem sieve plates interferes with the transport of photoassimilates of source leaves to the sink organs (Koh *et al*., 2012; Boava *et al*., 2017; Wang *et al*., 2017), resulting in excessive starch accumulation in leaf chloroplasts (Wang and Trivedi, 2013; Boava *et al*., 2017). Starch accumulation causes the disintegration of the chloroplast thylakoid system, producing the yellowing leaf mottle symptom (Schneider, 1968; Etxeberria *et al*., 2009). Consequently, other typical HLB symptoms occur, such as yellow shoots, hardened and small leaves, leaves showing zinc deficiency and corky veins, twig dieback, stunted growth, and tree decline (Bové, 2006; Wang and Trivedi, 2013).

Thus far, no source of resistance to HLB is known. However, the relative *Citrus* species *Poncirus trifoliata* does not present typical HLB symptoms, and multiplication of *C*Las remains low or nonexistent (Folimonova *et al*., 2009; Albrecht *et al*., 2012; Boava *et al*., 2015, 2017). Additionally, it is an important rootstock for citriculture because of its tolerance/resistance to *Phytophthora,* citrus tristeza virus and nematodes (Pang *et al*., 2007). Due to these characteristics, *P. trifoliata* and its hybrids have been highlighted as a possible source of tolerance/resistance to HLB. The hybrid population between *P. trifoliata* and *Citrus sunki* showed variability in response to *C*Las infection. Some hybrids were considered susceptible (*C*Las-positive and significant difference in starch levels), tolerant (*C*Laspositive, but no significant difference in starch levels) and resistant (*C*Las-negative and no difference in starch levels) (Boava *et al*., 2017).

We mapped the genomic regions associated with the expression analyses (eQTL) of *Citrus* callose synthase genes (*CscalS*) in the linkage groups of *C. sunki* and *P. trifoliata* genetic maps. Callose synthase genes encode the enzymes callose synthases (CalS), which are key elements for callose synthesis in different plant locations (Verma and Hong, 2001). In *Arabidopsis thaliana (At)*, 12 *calS* genes were identified and designated as *calS1*–*calS12* (Chen and Kim, 2009). In the *Citrus* genome, nine putative callose synthase (*calS*) genes could be found based on their amino-acid and DNA sequence similarities to *AtcalS* and they were named *CscalS2, CscalS3, CscalS5, CscalS7, CscalS8, CscalS9, CscalS10, CscalS11* and *CscalS12* (Granato *et al*., 2019).

Each *CalS* has a tissue-specific function (Ellinger and Voigt, 2014), and most are required for callose biosynthesis during pollen development (Jacobs *et al*., 2003; Enns *et al*., 2005; Töller *et al*., 2008). However, some callose synthases play important roles in response to pathogen infection (Dong *et al*., 2008; Luna *et al*., 2011). Particularly, *CalS7* has been demonstrated to be responsible for the synthesis of callose in sieve plates in *Arabidopsis* (Barratt *et al*., 2011; Wang *et al*., 2011).

Expression quantitative trait loci (eQTL) studies involve a direct association between genomic locations with gene expression levels (Nica and Emmanouil, 2013). eQTL evaluations using the *C. sunki* and *P. trifoliata* hybrids can be very important to understand the mechanisms involved in the development of HLB symptoms. Some regions associated with *CscalS* expression and, consequently, with callose deposition identified in this study can be considered potential targets for future citrus breeding programs aiming to obtain tolerance to HLB.

## Materials and Methods

### Plant material

The mapping population comprised 272 F1 hybrids resulting from crosses between *C. sunki* ex Tan (female parent) and *P. trifoliata* Raf. cv. Rubidoux (male parent). All the plants were propagated using buds grafted onto six-month-old Rangpur lime (*C. limonia* Osbeck). After six months, the plant scions were grafted on the opposite side of the primary stem, with two *C*Las-infected budwoods obtained from *C. sinensis* (L.) Osbeck cv. Pera plants, the identification of which was confirmed by qPCR. Infected budwoods were left on the plants, but shoots from these budwoods were eliminated upon sprouting. All the plants were kept in a greenhouse at Centro de Citricultura Sylvio Moreira of the Instituto Agronomico (IAC), Cordeiropolis/SP at an average temperature of 25 °C. The experiment comprised three biological replicates for each inoculated (*C*Las-infected budwood) and mock-inoculated (healthy budwood) genotypes.

For the gene expression assay and eQTL mapping, the leaves were collected from parental plants (*C. sunki* and *P. trifoliata*) and 72 hybrids from the F1 population, randomly selected, at 24 months after *C*Las inoculation.

### DNA extraction and molecular marker analysis

The leaves of 272 hybrids and the parental plants were collected at a similar age from four sides of the plants for DNA extraction. Five leaves were combined, and 200mg subsamples were lysed by grinding with two beads (3-mm diameter) in 2-mL microtubes at 30 Hz for 120 s in a TissueLyser II (Qiagen). DNA extraction was performed using the CTAB method (Murray and Thompson, 1980), and DNA quality and concentration were checked using a NanoDrop^TM^ 8000 spectrophotometer (Thermo Scientific, Waltham, Massachusetts, USA).

The hybrid population and parental plants were genotyped using SNP (single-nucleotide polymorphism) markers. The method used to obtain the molecular markers for *Citrus* using the DArT-seq platform was previously reported (Curtolo *et al*., 2017). Briefly, all the samples (272 hybrids and parents) were genotyped using *Pst*I and *Taq*I digestion and were sequenced on a HiSeq2000 DArT-seq device (Illumina Inc., San Diego, California, USA) at Diversity Arrays Technology Ltd. (DArT P/L, Canberra, Australia). The resulting sequences were aligned to the Clementine tangerine reference genome (https://phytozome.jgi.doe.gov/pz/portal.html). The DArT-seq technology detects both SNPs (Single Nucleotide Polymorphisms) and DArT-seq markers, which are based only on presence–absence (Raman *et al*., 2014). The molecular markers were represented in a dataset matrix where columns were the genotypes and rows were the markers. Parameters for quality control such as the call rate and reproducibility over 90% were adopted to select SNP markers for genetic mapping construction.

### Linkage maps

The linkage maps were obtained as previously described by Curtolo *et al*. (2018). All SNP loci that showed no deviation from the expected segregation were included in the analysis. The SNP molecular markers were coded according to Wu *et al*. (2002) in OneMap software (Margarido *et al*., 2007). Because this technology provides biallelic markers, three possible segregation patterns were expected: marker segregation for only the female parent (*C. sunki*) [ab x aa]; only for the male parent (*P. trifoliata*) [aa x ab]; and for both parents simultaneously [ab x ab]. The maps were constructed considering an LOD score = 8, and the maximum recombination fraction of 0.3. All the markers were aligned using BLASTn (Basic Local Alignment Search Tool) to the *C. sinensis* genome (https://citrus.hzau.edu.cn/) to establish the linkage groups because its assembly is based on pseudochromosomes while the Clementine genome is still based on scaffolds.

### RNA extraction and cDNA synthesis

We sampled the leaves from 72 hybrids and parent plants (*C. sunki* and *P. trifoliata*) both *C*Las and mock-inoculated (healthy plants). Leaves at a similar age were collected from four sides of the plants for RNA extraction. The samples were ground with liquid nitrogen, resulting in three microtubes with 100 mg for each genotype, consisting of three biological replicates per condition per genotype. Total RNA was extracted with lithium chloride (LiCl) using the protocol described by Chang *et al*. (1993) and adapted by Porto *et al*. (2010). The genomic DNA was eliminated using a DNase I, RNase-Free kit (Thermo Scientific, Waltham, Massachusetts, USA), according to the manufacturer’s recommendations, followed by purification with phenol-chloroform and ethanol precipitation. RNA quality was verified by agarose gel electrophoresis, and the RNA concentration was determined using a NanoDrop^TM^ ND-8000 spectrophotometer (Thermo Scientific, Waltham, Massachusetts, USA). cDNAs were synthesized from 1.0 µg of total RNA using Superscript III (200 U /µl) (Invitrogen, Carlsbad, California, USA) and oligo (dT) primers (dT12-18; Invitrogen) according to the manufacturer’s instructions. The obtained cDNA from the biological replicates was diluted in RNase-free water at the ratio of 1:50 and mixed, forming a pool of samples for each genotype to be analyzed in gene expression and eQTL mapping assays.

### Real-time quantitative PCR (RT-qPCR)

The cDNA pool from each genotype was diluted in RNAse-free water at the proportion of 1:25. The reaction comprised 6.0 µL of GoTaq qPCR Master Mix (Promega, São Paulo, Brazil), 2 µL of cDNA, 200 nM of each primer and water to a final volume of 10 µL. Amplifitions were carried out using two replicates for each sample with appropriate negative controls in the 7500 Fast Real-Time PCR System (Applied Biosystems, Foster City, California, USA) thermal cycler with the following conditions: 50 °C for 2 min; 95 °C for 10 min; 40 cycles of 95 °C for 15 s and 60 °C for 1 min.

The *CscalS* primers were based on Granato *et al*. (2019), and the endogenous controls (FBOX and GAPC2) were based on Mafra *et al*. (2012) (Table S1). The primer specificities were checked by melting curve analysis. Amplicons were sequenced using an ABI 3730 sequencer (Applied Biosystems, Foster City, CA, USA) and DyeTerminator chemistry to confirm their identities.

**Table 1:** Distribution of mapped SNP marker numbers and sizes (cM) for each linkage group in the *C. sunki* and *P. trifoliata* linkage maps.

	Linkage map			Linkage map	
	*C. sunki*			*P. trifoliata*	
	Number of	Size		Number of	Size
	markers	(cM)		markers	(cM)
LG 1	87	398.78	LG 1a	57	291.84
			LG1b	42	238.78
LG 2	73	348.65	LG 2	49	269.44
LG 3	44	185.48	LG 3	46	246.80
LG 4	48	311.13	LG 4	72	439.51
LG 5	113	530.91	LG 5a	47	332.32
			LG 5b	23	143.55
LG 6	61	293.06	LG 6a	31	156.96
			LG 6b	30	153.72
LG 7	73	358.47	LG 7	63	399.75
LG 8	11	63.68	LG 8	46	304.76
LG 9	61	364.84	LG 9	62	356.67
Total	571	2855	Total	568	3334.1

The amplification efficiency values (E) and Ct data were calculated for each RT-qPCR reaction using Real-time PCR Miner software (http://ewindup.info/miner/). The mean of the Ct values of the two technical replicates of each genotype was considered. Using these data, the relative quantification (fold change) was calculated using the 2^-ΔΔCT^ method (Livak and Schmittgen, 2001). The fold change was calculated using *C*Las-inoculated plants compared with the respective mock-inoculated plants with FBOX and GAPC2 as reference genes.

During RT-qPCR, 74 genotypes (72 hybrids, *C. sunki* and *P. trifoliata*) were separated in four plates (incomplete blocks). In each one, 18 genotypes and the parents were evaluated under mock-inoculated (healthy plants) and *C*Las-inoculated conditions. The experimental design used to evaluate the samples was an incomplete block design. The model used was as follows: Yij = mu + Bj + Gi + eij, where Yij corresponds to the gene expression of the i-th genotype evaluated in the j-th plate, mu is the model intercept, Bj is the fixed effect for plates, in which j varies from 1 to 4, Gi is the random effect of genotypes, in which i ranges from 1 to 74 and the genotypes 73 and 74 correspond to parents repeated along the four plates, and eij is the random residual effect. The function LME from package NLME of R software was used to analyze the mixed model and estimate the variance components.

### Gene expression profile and genetic parameter analyses

Fold-change values adjusted by the mixed model were used as inputs to the MeV (MultiExperiment Viewer) program v. 4.9 (http:// sourceforge.net/projects/mev-tm4/) to evaluate the gene expression profile. Evaluations were performed comparing the *CscalS* gene expression between the 72 hybrids and two parents (*C. sunki* and *P. trifoliata*) that were *C*Las inoculated and mock inoculated. The sets of genotypes with gene expression similarity were clustered using the hierarchical clustering method (HCL) and the Pearson correlation as the metric distance. The obtained values were graphically represented as a heatmap.

### eQTL mapping

The genetic linkage maps obtained for *C. sunki* and *P. trifoliata* were used for eQTL mapping. Relative gene expression values were analyzed using the composite interval mapping (CIM) strategy (Zeng, 1994), adapted to a single fullsib cross and implemented in the FullsibQTL package (Gazaffi *et al*., 2014) of the R software. Cofactor selection was performed using multiple linear regression analysis with a stepwise approach based on AIC (Akaike Information Criterion), similar to that performed by Souza *et al*. (2013) and Curtolo *et al*. (2018). The maximum number of selected cofactors was 20 with a window size of 1000 cM. The permutation test (Churchill and Doerge, 1994) was performed with 1000 replicates (*P*<0.05) to obtain the threshold (LOD score) to declare eQTL. However, the modification proposed by Chen and Storey (2006) was used. All genetic markers flanking an eQTL interval for *CscalS* were aligned with the Citrus reference genome (http://citrus.hzau.edu.cn/orange/) to check the presence of cis/trans eQTL using the BLASTn tool (https://blast.ncbi.nlm.nih.gov).

## Results

### C. sunki and P. trifoliata linkage maps

**Figure 1:**
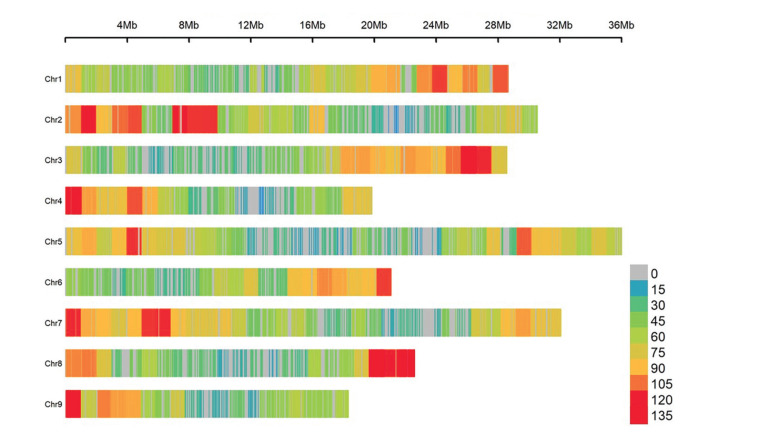
Density of markers in the chromosomes considering all markers resulting from the SNP technology from DArT-seq.

**Figure 2:**
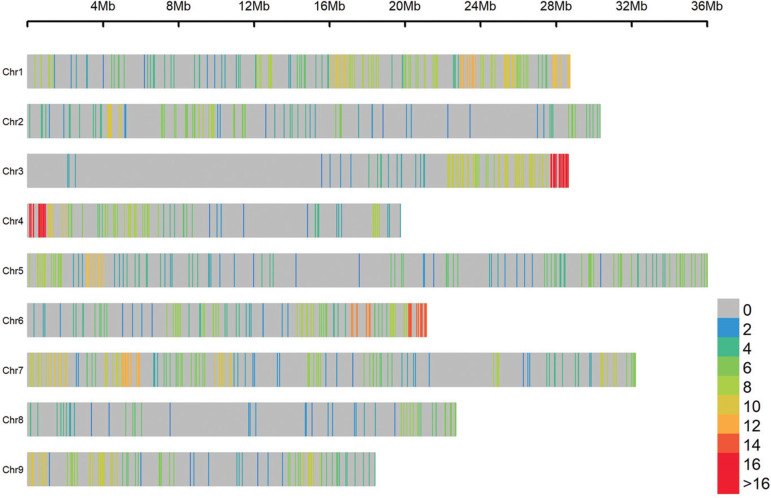
Density of markers in the chromosomes after considering a call rate < 90, missing calls in the parent genotyping for *C. sunki* and *P. trifoliata* and distortion segregation.

The linkage maps constructed were generated by SNP markers using 272 F1 hybrids from crosses between *C. sunki* and *P. trifoliata*. The F1 hybrids sampled were genotyped using 17,482 SNP markers, but 16,337 were excluded. The exclusion criteria for SNP markers were as follows: 2,437 SNP markers had a call rate < 90 (percentage of successfully scored individuals for an allele); 1,338 SNP markers showed distorted segregation; 6,914 SNP markers were homozygous for both parents; and 455 and 5,193 SNP markers were missing calls for *C. sunki* and *P. trifoliata*, respectively. The distribution of SNP markers before and after the exclusion is observed in Figures 1 and 2.

**Figure 3:**
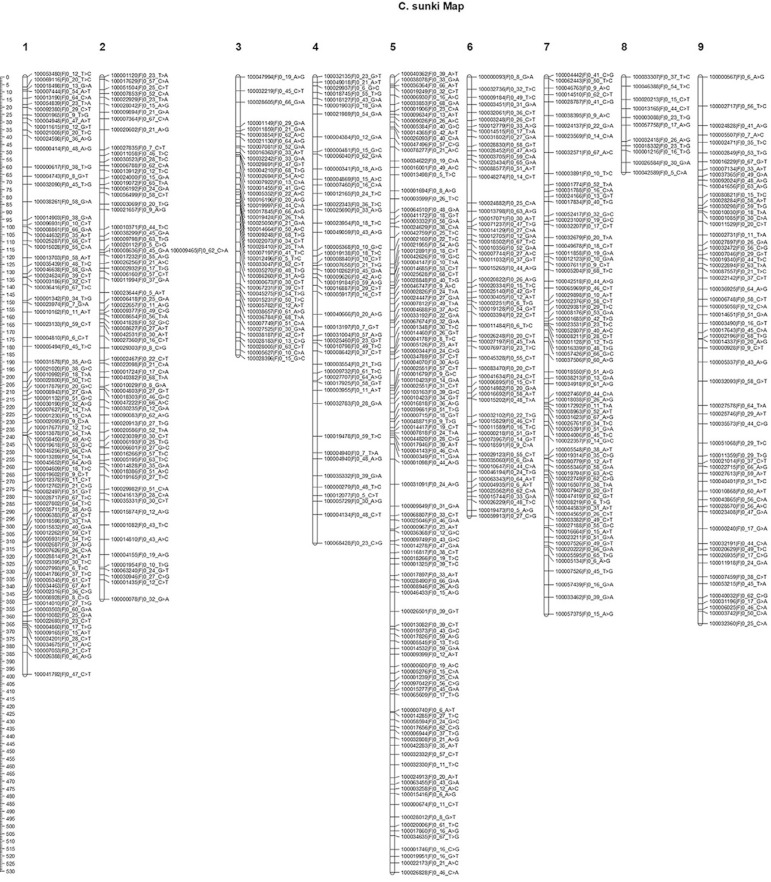
Linkage map of *C. sunki* using the pseudo-testcross strategy. Distribution of the 571 SNP markers on nine linkage groups of the *C. sunki* linkage map. X-axis represents linkage groups, and Y-axis indicates the genetic location (cM).

Regarding the remaining 1,145 SNP markers that showed a segregation ratio of 1:1, 571 SNP markers were polymorphic for the parent *C. sunki* and 574 for *P. trifoliata*. Initially, only 109 markers were common and polymorphic for both parents. On the other hand, these markers presented segregation deviation and therefore they were excluded. This fact resulted in an impossible integration of the linkage groups of both maps. The original approach proposed by Wu *et al*. (2002) results in a single integrated genetic map modeling the linkage phases between markers. We applied this methodology but analyzing as two separated data sets derived for each parent, similar to the pseudo-testcross strategy (Grattapaglia and Sederoff, 1994) and resulting in two separated maps. The *C. sunki* linkage map exhibited 571 loci and genomic coverage of 2,855 cM, distributed in nine linkage groups (LG) (Figure 3). The groups ranged from 63.68 (LG8) to 530.91 (LG5) cM. LG3 had the highest density of markers (4.21 cM between markers), and LG4 had the lowest density of markers (6.48 cM between markers).

**Figure 4:**
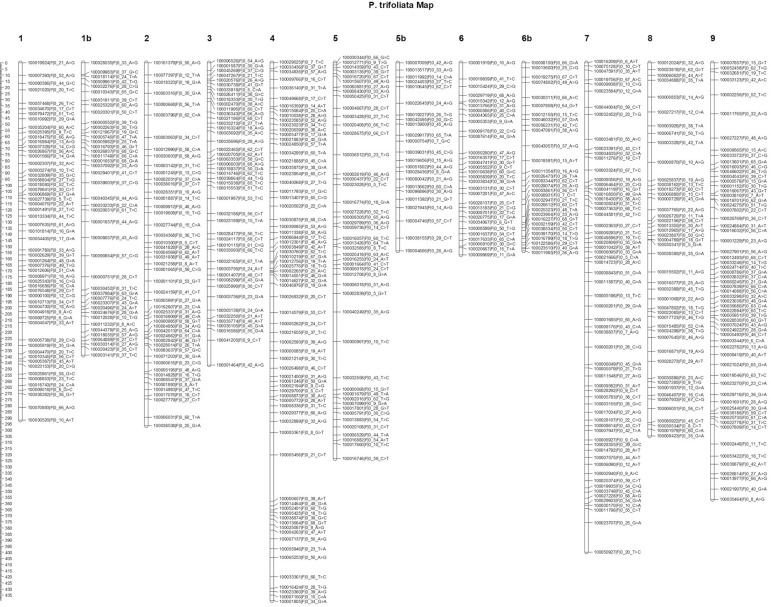
Linkage map of the *P. trifoliata* using the pseudo-testcross strategy. Distribution of the 568 SNP markers on the nine linkage groups of the *P. trifoliata* linkage map. X-axis represents linkage groups, and Y-axis indicates the genetic location (cM).

The *P. trifoliata* linkage map was constructed using 568 markers, and it had a genomic coverage of 3,334.1 cM, distributed in nine linkage groups (Table 1 and Figure 4). Only six SNP markers were not positioned on the map. Some linkage groups (LG1, LG5 and LG6) exhibited some large gaps. To avoid an overestimation of genomic coverage, we divided the linkage groups in subgroups adding the letters “a” and “b”. Based on the genomic information, the linkage groups were identified as LG1 to LG9 and ranged from 143.55 (LG5b) to 439.51 (LG4) cM. LG6a had the highest density of markers (5.06 cM between markers), and LG5a had the lowest density of markers (7.07 cM between markers). However, the molecular markers were compared with the genomic information, and some further information could be obtained (Table 2) e.g., 87 molecular markers were assigned to LG1 for the *C. sunki* map, among which 71 were correctly aligned with chromosome one, 13 were referred with an unassigned chromosome, and two markers were not aligned with a reference genome. Only one marker was wrongly assigned with other linkage groups, but the genomic information was assigned as chromosome one.

**Table 2:** Number of markers not aligned to the reference genome, aligned on the unassigned chromosome (UnChr), in another chromosome (X) or in the corresponding chromosome (Chr).

	*C. sunki*					*P. trifoliata*			
Linkage Groups	NotAlig	UnChr	X	Chr	Linkage Groups	NotAlig	UnChr	X	Chr
LG1	2	13	1	71	LG1a	1	13	1	42
					LG1b	0	4	0	38
LG2	1	14	2	56	LG2	0	8	3	38
LG3	0	0	0	44	LG3	0	2	2	42
LG4	1	3	8	36	LG4	0	4	14	54
LG5	0	30	5	78	LG5a	0	16	1	30
					LG5b	0	13	3	7
LG6	0	10	1	50	LG6a	0	5	3	23
					LG6b	0	0	0	30
LG7	1	3	0	69	LG7	0	8	0	55
LG8	0	0	0	11	LG8	0	5	7	34
LG9	1	15	4	41	LG9	0	15	6	41
Total	6	88	21	456	Total	1	93	40	434

* NotAlig represents all sequences that were not aligned to the reference genome; UnChr (unassigned chromosome) is a segment of the genome where none of the sequences are placed inpseudochromosomes; X represents all markers that were positioned in another chromosome which is not the one of the correspondences; Chr represents all markers that were aligned into corresponding chromosome.

A general view indicated that 456 (80%) of the markers from the *C. sunki* map and 434 (76%) of the markers from the *P. trifoliata* map were correctly grouped. Additionally, 88 (*C. sunki*) and 93 (*P. trifoliata*) molecular markers were assigned to an anonymous group (unassigned chromosome) in the reference genome i.e., they do not match any chromosome but the linkage approach provides extra information assigning along the genetic map. Only six markers of *C. sunki* and one marker of *P. trifoliata* were not assigned to the reference genome. Twenty-one markers of *C. sunki* and 40 markers of *P. trifoliata* were considered linked with groups that do not match genomic positions. In this case, the genomic position prevails to assign the markers to a specific group. Differences between genomic and map positions of markers may have resulted from false positives due to the multiple tests performed.

### Gene expression profile

**Figure 5:**
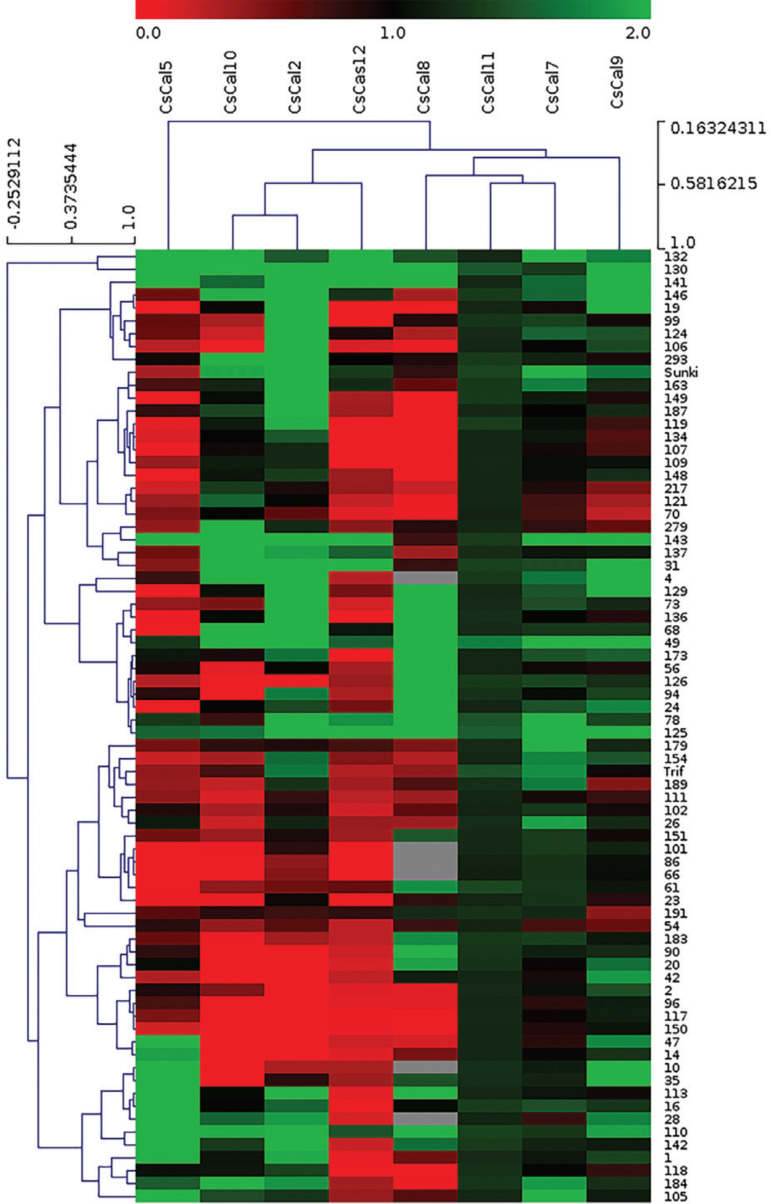
Heatmap of the gene expression profile by clustering analysis between the eight *CscalS* genes evaluated using the 74 genotypes (72 hybrids and the parent plants *P. trifoliata* and *C. sunki*). The heatmap was made using fold-change normalized data as inputs to the MeV (MultiExperiment Viewer) program v. 4.9 (http://sourceforge.net/projects/mev-tm4/). The names of genes and gene hierarchical clusters are shown at the top. Fold-change expression values ranged from green (highest expression) to red (lowest expression). The sample names (74 genotypes) are shown on the right side, while the sample hierarchical cluster is shown on the left side.

According to the heatmap (Figure 5), the parental *C. sunki* and 43% of hybrids plants showed a predominantly green overall expression pattern, indicating that genotypes 132, 130, 141, 146, 19, 99, 124, 166, 293, 163, 149, 187, 119, 134, 107, 109, 148, 217, 121, 70, 279, 143, 137, 31, 4, 129, 73, 136, 68, 49, 173, and the parental *C. sunki* showed upregulation of *CscalS* gene expression compared with the *C*Las-infected plants and healthy controls. On the other hand, most of the genotypes (57%) i.e., hybrids 56, 126, 94, 24, 78, 125, 179, 154, 189, 111, 102, 26, 151, 101, 86, 66, 61, 23, 191, 54, 183, 90, 20, 42, 2, 96, 117, 150, 47, 14, 10, 35, 113, 16, 28, 110, 142, 1, 118, 184, 105, and the parental *P. trifoliata* exhibited downregulation in the expression of *CscalS* genes compared with that in the *C*Las-infected plants and heathy controls.

In the same analysis, the parental *P. trifoliata* showed upregulated expression of *CscalS2* and *CscalS7*, while *CscalS11* and the parental *C. sunki* displayed upregulated expression of *CscalS2, CscalS7, CscalS9, CscalS10, CscalS11* and *CscalS12*. Regarding the hybrids, it is possible to observe that regulation of the analyzed *CscalS* genes was very different among them. The expression of *CscalS2* and *CscalS7* was upregulated in most genotypes, including the parental *C. sunki* and *P. trifoliata. CscalS9* and *CscalS10* also demonstrated upregulation in 53 genotypes. *CscalS5* and *CscalS12* were revealed to be largely down-regulated in the genotypes. The expression of *CscalS11* presented upregulation in all the genotypes analyzed, and *CscalS8* was upregulated in 27 genotypes.

The heatmap (Figure 5), based on the comparative analysis performed by hierarchical clustering (HCL) of *CscalS* genes and the 72 hybrids plus their two parents (*C. sunki* and *P. trifoliata*) allowed the grouping of genes and related genotypes. Additionally, Pearson’s correlation was used as a metric distance to obtain the best intra and inter-variable grouping possible. The genotypes were separated into eight subgroups distributed into three main clusters. The parent *P. trifoliata* was internally clustered with the genotypes 154 and 189, while the parent *C. sunki* was clustered together with the genotypes 163 and 149. Both parent clusters were grouped with the remaining genotypes to form a larger main cluster.

The genes were separated into three clusters. The first cluster was formed by *CscalS2, CscalS10* and *CscalS12*, the second cluster was formed by *CscalS7, CscalS8, CscalS9* and *CscalS11*, and a third one was formed only by *CscalS5*.

The adjusted values of the *CsCalS* relative gene expression from the F1 hybrids were used to calculate the genetic parameters (heritability, variance, and coefficient of variation). The genotypic variance (Vg) ranged from 0.11 to 40.81, expressed as the genotypic variation coefficient (CVg) that varied from 26.11 to 369.23% (Table 3). Phenotypic variance (Vf) estimates varied from 1.37 to 41.22, and the highest values were obtained for the genes *CscalS8* (41.22) and *CscalS*12 (15.95). High values of heritability (h^2^) for the studied callose synthase genes were observed, with the exception of *CscalS*11 (6.00), indicating that, for this gene, the genotypic variance was proportionally lower than the environmental variance.

**Table 3:** Estimates of genotypic and phenotypic variances, heritability and coefficients of variation for gene expression.

Genes	Vg	Vf	h^2^ (%)	CVr (%)	CVg (%)
*CscalS2*	7.94	8.44	94.07	33.11	137.04
*CscalS5*	11.33	11.83	95.77	44.75	213.03
*CscalS7*	0.80	1.55	51.61	61.48	64.81
*CscalSH*	15.48	15.95	97.05	32.18	184.71
*CscalS9*	1.23	1.37	89.78	24.94	73.93
*CscalS10*	40.81	41.22	99.00	37.01	369.26
*CscalSll*	0.11	1.69	6.00	98.97	26.11
*CscalS12*	1.42	1.62	87.65	72.13	192.19

Vg = genotypic variance; Vf= phenotypic variance; h^2^ = heritability; CVr = coefficient of variation of the residue; CVg = coefficient of variation of the genotype.

### eQTL mapping

It was possible to detect eQTL in response to infection caused by *C*Las using the *C. sunki* and *P. trifoliata* linkage maps and gene expression profiles from the relative expression values (fold change) of *CscalS* genes evaluated in the 72 hybrids.

**Figure 6:**
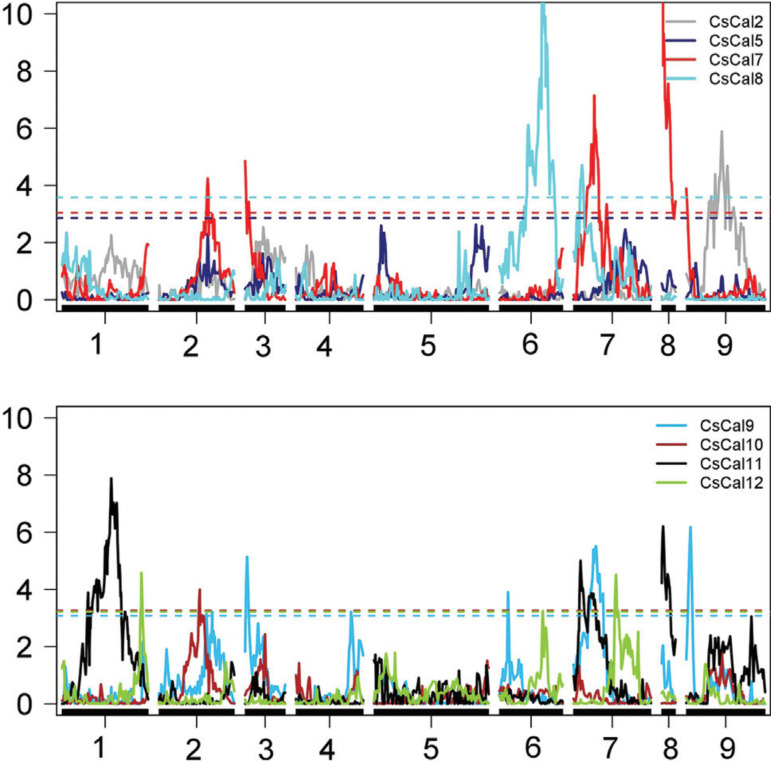
Detection of eQTL in the *C. sunki* linkage map related to the expression of the *CscalS* genes evaluated. Y-axis: LOD; X-axis: distance incentiMorgans; the dashed lines represent threshold values obtained using 1000 replicates.

Considering the *CscalS* expression profile, 18 eQTL were mapped in the *C. sunki* linkage map, and the LOD scores of the eQTL ranged from 3.22 to 17.87 (Figure 6 and Table 4). All eQTL detected showed a 1:1 segregation pattern, and they were mapped in all linkage groups, except LG5. One eQTL was detected for *CscalS2* on LG9; five eQTL for *CscalS7* were detected on LG2, LG3, LG7, LG8 and LG9; two eQTL for *CscalS8* were detected on LG6 and LG7; six eQTL for *CscalS9* were detected on LG2, LG3, LG4, LG6, LG7 and LG9; one eQTL for *CscalS10* was detected on LG2; and three eQTL for *CscalS12* were detected on LG1, LG6 and LG7. It was not possible to detect eQTL for *CscalS5* and *CscalS11*. The phenotypic variance values (R^2^) explained by the eQTL mapped varied from 0.49% to 20.18%. The eQTL detected for *CscalS7* on LG8 exhibited the highest R^2^ using the *C. sunki* map (20.18%). Together, the five eQTL for *CscalS7* explained 53.12% of the phenotypic variation; thus, *CscalS7* had the highest percentage of the phenotypic variation explained by the eQTL mapping. The highest number ofeQTL was detected for *CscalS9* (six eQTL), and, overall, they represented 30.38% of the phenotypic variation. The three eQTL were identified for *CscalS12*, explaining 30.46% of the phenotypic variation.

**Table 4:** eQTL mapping for *CscalSl, CscalSl, CscalS8, CscalS9, CscalS10, CscalSH* in *C. sunki* linkage map.

Genes	SNP Markers	Genome position	LG	cM	Lod-Score	Additive Effect	_R_2
***CscalS2***	100003490|F|0_16_G > T	ChrUn,1142507	9	164.32	5.92	0.78	12.31
*CscalS7	100090083|F|0_62_A > G	Chr2,7755160	2	225.47	4.25	-1.01	0.82
*CscalS7	100047994|F|0_19_A > G	Chr3,19075229	3	0.00	5.06	1.79	7.42
*CscalS7	100023100|F|0_19_G > C	N/D	7	96.17	7.19	2.08	17.99
***CscalS7***	100033307|F|0_37_T > C	Chr8,19898080	8	0.00	17.87	3.05	20.18
*CscalS7	100000567|F|0_6_A > G	Chr9,17314839	9	0.00	3.90	-1.16	6.71
***CscalS8***	100041634|F|0 24 C > T- 100006895|F|0_15_C > T	Chr6,15796184-15817077	6	203.00	11.50	-0.30	10.91
***CscalS8***	100023569|F|0_14_C > A	Chr7,1786000	7	39.42	4.71	0.21	5.29
*CscalS9	100006193|F|0_25_T > G	Chr2,7224068	2	246.57	3.22	-0.33	7.11
*CscalS9	100032219|F|0_45_C > T	Chr3,19755543	3	9.20	5.17	0.36	3.34
***CscalS9***	100004940|F|0_48_A > G	Chr7,3129395	4	254.71	3.25	-0.27	3.31
***CscalS9***	100031802|F|0_27_G > A	Chr6,5552031	6	39.72	3.91	-0.30	1.23
*CscalS9	100032207|F|0_17_C > T- 100032679|F|0_20_T > A	Chr7,6721626-7216583	7	103.00	5.51	0.38	6.04
*CscalS9	100002717|F|0_56_T > C	ChrUn,50210454	9	19.40	6.18	-0.39	9.35
***CscalSIO***	100002467|F|0_22_C > T	Chr2,13556907	2	189.02	4.01	-0.52	0.49
***CscalSI2***	100001230|F|0_15_C > A	Chr1,16786655	1	367.38	4.59	0.42	7.57
***CscalSI2***	100024137|F|0_22_G > A	Chr7,1434034	6	200.00	3.26	-0.27	11.46
***CscalSI2***	100046388|F|0_54_T > C	Chr8,20056662	7	196.59	4.51	-0.43	11.43

SNP markers = flanking markers; LG = Linkage Group; cM = position; R^2^ = explained phenotypic variation;

* = hot spot

The colocalization of eQTL may suggest the existence of hot spots. eQTL for *CscalS7* and *CscalS9* could be observed on LG2, LG3, LG7, and LG9 separated by 21.00, 9.20, 6.83, and 19.40 cM, respectively. Considering the 18 eQTL identified in the *C. sunki* map, eight were clustered in four different hot spots.

**Figure 7:**
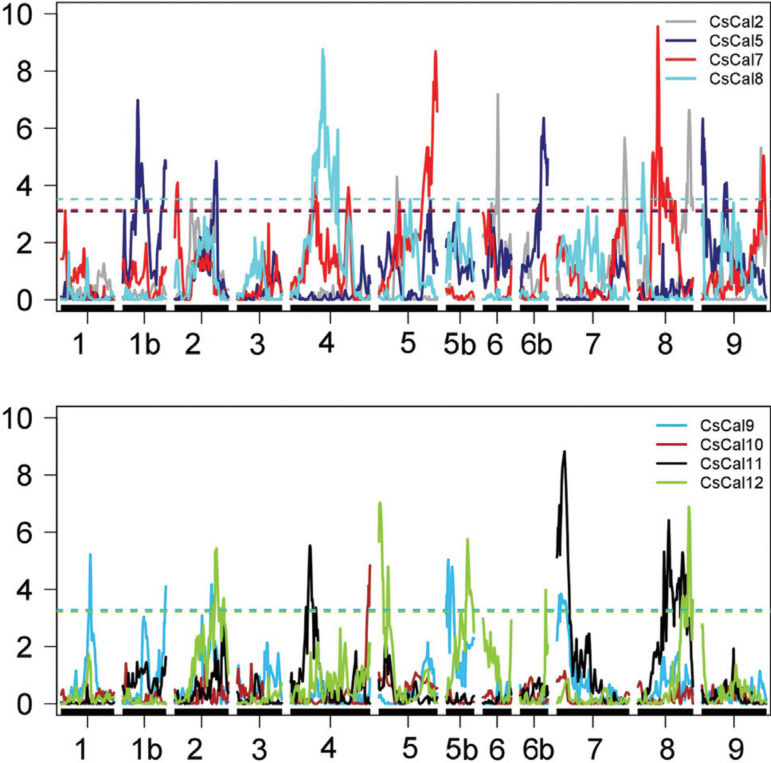
Detection of eQTL in the *P. trifoliata* linkage map related to the expression of the *CscalS* genes evaluated. Y-axis: LOD; X-axis: distance in centiMorgans; the dashed lines represent threshold values obtained with 1000 replicates.

In the *P. trifoliata* linkage map, it was possible to map 34 eQTL (Figure 7 and Table 5): eight eQTL for *CscalS2* were distributed on LG2, LG4, LG5, LG6, LG7, and LG8; seven eQTL for *CscalS5* were distributed on LG1b, LG2, LG5, LG7, LG9; seven eQTL for *CscalS7* were distributed on LG2, LG4, LG5, LG8, LG9; two eQTL for *CscalS8* were distributed on LG4 and LG8; five eQTL for *CscalS9* were distributed on the LG1, LG1b, LG2, LG5b, LG7; and five eQTL for *CscalS12* were distributed on LG2, LG5, LG5b, LG7, LG8. No eQTL was identified for either *CscalS10* or *CscalS11*.

**Table 5:** eQTL mapped for *CscalSl, CscalS5, CscalSl, CscalS8, CscalS9, CscalSH* in the *P. trifoliata* linkage map.

Genes	SNP Markers	Genome Position	LG	cM	Lod-score	Additive Effect	R^2^
***CscalS2***	100001245|F|0 13 C > G- 100002031|F|0_37_C> A	Chr2,11496268- 12594168	2	92.00	3.54	0.70	3.68
*CscalS2	100025331|F|0_31_A > G	Chr2,9722118	2	206.42	3.40	0.69	2.13
*CscalS2	100005456|F|0_21_C > T	Chr7,11995806	4	320.42	3.20	-0.59	0.4
***CscalS2***	100023028|F|0_5_T > C	Chr5,6320268	5	99.56	4.30	0.81	8.32
***CscalS2***	100004741|F|0_30_G > T	Chr6,7357918	6	83.01	7.19	1.00	12.47
***CscalS2***	100023707|F|0_25_G > A	Chr7,1472171	7	375.76	5.67	-0.80	4.22
*CscalS2	100006051|F|0 56 C > T- 100080922|F|0_45_C > T	Chr8,158039	8	284.00	6.64	-0.87	9.27
*CscalS2	100038879|F|0_42_A > T	Chr9,168999717	9	327.91	5.32	1.00	9.14
***CscalS5***	100037092|F|0_33_A > G	Chr1,24513911	1b	83.13	6.98	-0.89	2.29
*CscalS5	100020423|F|0_35_C > T 100003141|F|0_37_T > C	ChrUn,62887483- 62915479	1b	235.00	4.88	-0.75	2.55
*CscalS5	100028014|F|0_26_T > A	Chr2,8399713	2	229.25	4.83	1.15	8.67
***CscalS5***	100005791|F|0_30_C > G	ChrUn,38031312	5	285.46	3.49	-0.79	3.33
***CscalS5***	100026612|F|0_60_C > T	Chr6,19905462	7	130.47	6.35	0.79	8.18
***CscalS5***	100052458|F|0_62_T > G	Chr9,752864	9	5.10	6.32	0.95	17.15
***CscalS5***	100016032|F|0_56_C > A	Chr9,7003215	9	136.41	4.15	0.78	3.40
***CscalS7***	100018323|F|0_19_G > A	ChrUn,32178022	2	15.96	4.09	-0.88	4.69
***CscalS7***	100011338|F|0_50_A > G	Chr4,6197839	4	138.66	4.09	-0.92	4.13
*CscalS7	100005456|F|0_21_C > T	Chr7,11995806	4	320.42	3.96	-0.90	3.24
*CscalS7	100016774|F|0_18_G > A	Chr5,7632775	5	114.08	3.43	-0.92	4.37
***CscalS7***	100017660|F|0 10 T > C- 100016746|F|0_59_C > T	Chr5,27887080- 29928945	5	314.00	8.68	-1.69	10.85
***CscalS7***	100000729|F|0_43_G > A	Chr6,13766295	8	110.29	9.55	-2.09	22.63
*CscalS7	100013977|F|0 66 A > G- 100021907|F|0_40_G > A	Chr9,18067045	9	342.00	5.04	-1.09	5.7
***CscalS8***	100014627|F|0 32 G > A- 100046976|F|0_19_G > A	Chr4,7777178	4	178.00	8.75	-0.26	8.88
***CscalS8***	100000853|F|0_14_A > G	ChrUn,88833722	8	27.94	4.78	-0.17	4.09
***CscalS9***	100001264|F|0_48_G > A	ChrUn,22371945	1	161.86	5.21	-0.36	7.64
*CscalS9	100003141|F|0_37_T > C	ChrUn,62915479	1b	238.77	4.09	0.29	4.87
*CscalS9	100162807|F|0_23_C > A	Chr2,9832235	2	203.43	4.17	0.24	0.8
***CscalS9***	100011992|F|0_14_C> A	ChrUn,4717070	5b	11.66	5.03	-0.30	4.83
***CscalS9***	100023584|F|0_12_G > A	Chr7,31022976	7	22.95	3.84	0.30	5.49
*CscalS12	100083637|F|0_57_G > C	Chr2,8444059	2	230.56	5.42	0.31	5.48
***CscalS12***	100003135|F|0_39_G > T	Chr5,8444059	5	5.83	7.03	-0.35	5.98
***CscalS12***	100021945|F|0_14_A > G	Chr5,33708464	5b	118.36	5.76	0.30	3.72
***CscalS12***	100002159|F|0_42_C > T	Chr6,21087431	7	141.72	3.99	-0.33	7.15
*CscalS12	100006051|F|0 56 C > T	Chr8,2038979	8	282.42	6.89	-0.33	7.6

SNP markers = flanking markers; LG = Linkage Group; cM = position; R^2^ = explained phenotypic variation;

* = hot spot

Overall, R^2^ varied from 0.4 to 22.63%, the LOD score ranged from 3.21 to 9.56 and all segregated in a 1:1 fashion. Considering the eQTL mapping for *P. trifoliata*, eQTL for *CscalS7* had the highest R^2^ (22.63%) and, when the seven eQTL were considered together, they summed the highest R^2^ (55.61%). The region with the lowest R^2^ was identified for *CscalS2*, explaining only 0.4% of the phenotypic variation.

*CscalS2* had the highest number of regions detected in this study. Thirty-nine percent of the phenotypic variation were explained by the eight eQTL detected for *CscalS2*. Five other markers were associated with *CscalS8*, and, overall, they summed an R^2^ of 39.62%. Two eQTL detected for *CscalS2* and *CscalS12* were overlapped. They were located on LG2 approximately 203-206 cM and further on two eQTL that were overlapped for *CscalS5* and *CscalS12* (230 cM). Another overlap eQTL for *CscalS*5 and *CscalS*9 was found on LG1b. The co-location of eQTL was detected for *CscalS2* and *CscalS12* on LG8, separated by 2.42 cM. Three overlap loci were identified between *CscalS2* and *CscalS7*: the first on LG4, the second separated by 14 cM on LG5 and the last on LG9 distant by 14 cM.

The existence of eQTL was noticed for the same *CsCalS* and LG in *C. sunki* and *P. trifoliata* maps. In both maps, eQTL were detected for *CscalS2* on LG9, *CscalS7* on LG2, *CscalS7* on LG8 and LG9, *CscalS9* on LG2 and LG7 and *CscalS12* on LG7. It is worth highlighting that the major eQTL identified in the *C. sunki* and *P. trifoliata* maps was positioned in the same linkage group (LG8).

Genomic information, such as the physical position, is not always accessible for *CscalS*; thus, inferring whether *cis* or *trans* eQTL exist becomes a challenge. Only the physical position is available for *CscalS2* (Chr 7), *CscalS5* (Chr 1), *CscalS7* (Chr 7), *CscalS8* (Chr 5) *CscalS10* (Chr 5), and *CscalS11* (Chr 2) (Granato *et al*., 2019). However, there is no eQTL close to the genes, suggesting the presence of epistatic eQTL or *trans* eQTL. In the cases of *CscalS9* and *CscalS12*, for which the physical locations are not described, an inference between *cis* and *trans* is not feasible.

## Discussion

The hybrid population obtained from *C. sunki* and *P. trifoliata* crossing was genotyped using 17,482 SNP markers. However, the *C. sunki* and *P. trifoliata* genetic linkage maps were constructed using 571 and 568 representative SNP markers, respectively. Although a high number of SNP markers has been generated by genotyping using sequencing technology, many markers were excluded from the analysis due to the drawback of many lines being multiplexed during sequencing. Moreover, 1,338 SNP markers did not show the expected segregation. Deviations from the segregation can be the result of crosses among different genera (*Citrus* and *Poncirus*), as previously reported (Curtolo *et al*., 2018). The SNP marker exclusion resulted in a low number of polymorphic markers. We believe that monomorphic markers are often generated by technical and biological reasons. Genotyping technology with library construction, read depth, and data handling are possible causes of the presence of noninformative markers. Additionally, we should consider the limited population size as a possible explanation of monomorphic marker presence because the number of genotyped individuals determines the chance to detect recombinant loci. A large ratio of monomorphic markers has been reported as a disadvantage of high-throughput genotyping (Shimada *et al*., 2014; Guo *et al*., 2015; Yu *et al*., 2016a; Curtolo *et al*., 2017; Imai *et al*., 2017). It should be noted that the crossing between two parents from different genera contributes to few marker polymorphic at the same time for both parents i.e., SNPs are not as old as that required for being shared by *C. sunki* and *P. trifoliata* because SNPs are conservative markers. This corroborates the idea that both parents are not genetically related and explains why two maps were obtained, one for each parent. Previously, Curtolo *et al*. (2018) used dominant markers such as DArTseq and obtained loci shared by *C. sunki* and *P. trifoliata*; however, the number of markers was not sufficient to enable information integration from both parents.

SNPs have been considered the most attractive markers to obtain genetic mapping, and they can be genotyped in parallel assays at low costs in marker-assisted breeding (Bertioli *et al*., 2014). There are six genetic maps for *Citrus* using SNP markers (Ollitrault *et al*., 2012; Xu *et al*., 2012; Guo *et al*., 2015; Yu *et al*., 2016a; Imai *et al*., 2017; Huang *et al*., 2018). However, this study is the first to demonstrate a linkage map for *Citrus* using SNP markers obtained from DArT-seq technology.

*C. sunki* and *P. trifoliata* linkage maps showed SNP markers distributed in nine linkage groups, corresponding to the haploid number of chromosomes of citrus. In both maps, few SNP markers were positioned in a different chromosome where most of the markers were located (Table 2). The difference in the marker position can be caused by the assembled difference between the species used in the reference genome and constructed linkage maps. The establishment of the marker position that has been grouped in the unassigned chromosome (UnChr) is a contribution of the present work. Furthermore, it could help update the *Citrus sinensis* genome, as previously reported by Curtolo *et al*. (2017). In the *P. trifoliata* map, some linkage groups were separated into “a” and “b” groups to avoid an overestimation of the genomic coverage. Nevertheless, the map and some groups of *P. trifoliata* are larger than those designed for *C. sunki*. Other authors also showed differences among linkage group sizes (Chen *et al*., 2008; Huang *et al*., 2018). The recombination rate, which is used to obtain the maps, is distinct between females and males, both in plants and animals (Lorch, 2005). Ollitrault *et al*. (2012) and Huang *et al*. (2018) noticed that the size of male genetic maps is usually larger than that of female genetic maps. It corroborates the linkage maps obtained in this study, because *C. sunki* was the female parent and *P. trifoliata* was the male parent of the crossing, generating the studied hybrid population.

The presented linkage maps are a substantial resource for future studies of *Citrus*. The parents and hybrids used for the analyses revealed many important characteristics for citriculture. For example, both parents are important rootstocks, and *C. sunki* has high vigor and good fruit yield, as well as tolerance to Tristeza, citrus blight disease and salinity (Castle *et al*., 1993). *P. trifoliata* is immune to citrus tristeza virus and resistant to nematodes, although it has low tolerance to drought (Passos *et al*., 2006). *P. trifoliata* was also reported to be more tolerant to HLB because it does not show starch accumulation in leaf chloroplasts and does not show typical HLB symptoms, unlike *C. sunki* (Boava *et al*., 2017).

The excessive accumulation of starch in *Citrus* leaves during *C*Las infection has often been associatedwith photo-assimilate transport disturbance (Koh *et al*., 2012; Boava *et al*., 2017; Wang *et al*., 2017). The reduction of photo-assimilate transport of leaf sources to the sink organs results from deposition of callose and phloem proteins (PP2) in the phloem of infected plants (Koh *et al*., 2012; Wang and Trivedi, 2013; Boava *et al*., 2017). Callose is synthetized by the callose synthase enzymes (*CalS*), whose activity is highly regulated by pathogen infection (Yu *et al*., 2016b; Granato *et al*., 2019). In this study, the expression of all evaluated *CscalS* was regulated in *C*Las-infected citrus leaves, demonstrating that multiple callose synthase genes can be expressed in the same organ (Dong *et al*., 2008; Granato *et al*., 2019). Most of the genotypes analyzed (57%), including the parental *P. trifoliata*, showed *CscalS* gene expression downregulation comparing the *C*Las-infected plants and heathy controls. On the other hand, the parental *C. sunki* and 43% of the genotypes showed upregulation of *CscalS* gene expression after *C*Las infection.

The *CscalS*2 gene was upregulated in many genotypes, including the parental *C. sunki.* CalS2 has not been characterized yet. However, in *Arabidopsis*, it shares high homology (92% identity) with CalS1, suggesting that a gene duplication event may have occurred, and it is possible that the two genes encoding both enzymes are functionally redundant (Hong *et al*., 2001). *CscalS2* upregulated expression in *C. sunki* and hybrids may indicate that this gene plays an important role in callose accumulation, as a strategy to alter plasmodesma permeability under *C*Las infection because it occurs in *Arabidopsis* rosette leaves after salicylic acid (SA) and *Hyaloperonospora arabidopsis* infection (Cui and Lee, 2006; Dong *et al*., 2008).

*CscalS7* has been demonstrated to be responsible for callose deposition specifically in the phloem sieve tubes (Barratt *et al*., 2011; Xie *et al*., 2011). *CscalS7* was upregulated in *P. trifoliata* in *C*Las-infected plants. However, upregulation was lower than that observed for *C. sunki* (Table S2). The *CscalS7* gene was also upregulated in 49 other genotypes. The lower expression value of *P. trifoliata* can be due to its tolerance to HLB, or callose deposition in *P. trifoliata* does not cause hypertrophy of the phloem parenchyma cells and collapse of the sieve tube elements (STE) because it occurs in *C. sunki* (Folimonova *et al*., 2009; Koh *et al*., 2012). As previously shown for the HLB pathosystem (Granato *et al*., 2019) and grapevine-resistant cultivar *Vitis amurensis* `Shuanghong׳ infected with *Plasmopara viticola* (Yu *et al*., 2016b), *calS7* upregulation after infection indicates that callose deposition specifically at phloem sieve tubes occurs to block the flow of the pathogens, which probably occurred in *C. sunki, P. trifoliata* and their hybrids.

Other *CscalS* also presented upregulation in the analyzed genotypes, such as *CscalS9, CscalS10,* and *CscalS12.* CalS9 and CalS10 functions have been more related to gametophyte development (Töller *et al*., 2008) than the plant defense response. Nevertheless, the biological role of *calS12* has been well studied in the stress and pathogen response (Nishimura *et al*., 2003; Dong *et al*., 2008; Luna *et al*., 2011; Ellinger and Voigt, 2014). For example, *calS12* is required for callose deposition in cell wall thickenings at the sites of fungal pathogen attack during powdery mildew infection (Dong *et al*., 2008). Additionally, Granato *et al*. (2019) also demonstrated that, in *C. sinensis*, at 360 days after infection, *CscalS12* was significantly upregulated in HLB-positive plants. These results indicate that *CscalS12* is also likely involved in callose deposition after *C*Las infection. Because all callose synthase genes showed regulation of expression after *C*Las infection, it is possible that multiple *CscalS* work like a complex in the phloem sieve tubes, causing callose accumulation after pathogen attack (Granato *et al*., 2019).

Some genotypes studied in this work were classified by Boava *et al*. (2015) as tolerant or susceptible, based on the starch accumulation and titer of *C*Las. Genotypes 19, 119, 124, 217 and *C. sunki* were previously classified as susceptible, and our results showed upregulation of *CscalS2, CscalS7* and *CscalS11* expression and downregulation of *CscalS5* and *CscalS8* expression after *C*Las infection. Additionally, genotypes 66, 102 and *P. trifoliata*, classified by Boava *et al*. (2015) as tolerant, presented the same expression pattern of susceptible plants (19, 119, 124 and 217), except for *CscalS2*. Thus, making a connection between the expression values and level of tolerance or susceptibility is unlikely.

To find an association between the quantification of *CscalS* transcripts and allelic status of a genome region, we mapped the genomic regions associated with *CscalS* expression analysis in the linkage groups of *C. sunki* and *P. trifoliata* genetic maps. These genomic regions, referred to as eQTL, are important to understand the *C*Las-host plant interaction and mechanisms of tolerance and response to HLB.

It was possible to identify eQTL for *CscalS2, CscalS7, CscalS8, CscalS9,* and *CscalS12* for both parents, although *P. trifoliata* is tolerant and does not exhibit callose deposition or starch accumulation after *C*Las infection (Boava *et al*., 2017). In contrast, no eQTL was found for *CscalS11* due to the low variation of expression data among *C*Las-infected and healthy plants. Based on the estimation of the genetic parameters, *CscalS11* presented low heritability, indicatings that the environment has great influence on this gene. Presumably, the regions that control the genetic variability for *CscalS11* were not segregated in the study population, making it impossible to detect eQTL. The presence of important loci in homozygosity in both parents is a likely explanation for the absence of segregation for *CscalS11*.

Considering all eQTL mapped for the *CscalS7* gene, they explained the highest percentage of the phenotype variation between *C*Las-infected and healthy plants. Thus, it is possible to state that *CscalS7* is the most affected evaluated gene after *C*Las infection and is the most responsible for callose synthesis in the *C*Las-infected plants.

Other evaluated genes were also affected by *C*Las infection. eQTL were mapped for *CscalS2, CscalS7, CscalS8, CscalS9, CscalS10, and CscalS12* in the *C. sunki* map and for *CscalS2, CscalS5, CscalS7, CscalS8, CscalS9,* and *CscalS12* in the *P. trifoliata* map. In *C. sunki*, more than 44% of the eQTL observed were overlapped, characterizing hot spots. Thus, there are genomic regions that regulate the expression of more than one *CscalS* gene e.g., the main region on LG6 (200-203 cM) probably modulates *CscalS8* and *CscalS12* expression. In the *P. trifoliata* map, seven regions were considered hot spots and another 20 regions were mapped. Almost half of eQTL detected for *CscalS2* and *CscalS7* were overlapped. These regions and the other hot spots detected could probably be related to callose synthesis after *C*Las infection.

Apparently, both parents contribute to the response of the callose synthase gene expression because many eQTL were observed in the same chromosome for *CscalS* in both maps. Based only on the SNP markers, it is hard to establish a direct correlation between the maps. However, comparing the eQTL for *CscalS*, an important region was verified for *P. trifoliata* on chromosome 8 that could influence the expression of *CscalS7* in plants affected by HLB.

The data sets obtained in this study revealed that it is not possible to determine whether the eQTL detected for *CscalS* in both maps represent the same genomic regions. Future studies should be considered to integrate the information from different materials.

Some eQTL can alter the expression of other genes located near them (*cis*-eQTL), explaining the variation of gene expression in the chromosomal region where the gene was found. On the other hand, other eQTL can regulate the expression of genes located distant from them (*trans*-eQTL), representing an effect of genetic polymorphisms that are located in other regions of the genome (Lima *et al*., 2018). The position of *calS* was confirmed to be in the *Citrus sinensis* genome (http://citrus.hzau.edu.cn/orange/); however, some genes did not have a defined position on pseudochromosomes because *CscalS9* and *CscalS12* were grouped on UnChr. Thus, for some cases, it was appropriate to determine whether the eQTL identified altered expression of nearby transcripts (*cis*-eQTL) or remote transcripts (*trans*-eQTL), usually on different chromosomes. Four SNP markers from the *P. trifoliata* map associated with *CscalS2, CscalS5* and *CscalS7* were exclusively on the same chromosome as the genes, although they have been classified as *trans*-eQTL, because they are separated by more than 1 kb. Based on this investigation, we concluded that it is necessary to allocate *CscalS9* and *CscalS12* on the nine *Citrus* pseudochromosomes to make it possible to identify *cis*-eQTL. None of the SNP markers associated with *CscalS* expression was located on the region where the gene was found; therefore, probably all of the eQTL described in this study have an epistatic effect. The nonidentification of *cis*-eQTL could be due to two reasons for *CscalS* that has a physical position in the genome. First, the effect of some eQTL could be relatively low, hindering its mapping. Second, the polymorphism could be homozygous, causing possible variation in *cis*, such as promoters or enhancers (or other gene regulatory agents), with no segregation of the loci in the progeny.

Considering that *CscalS9* and *CscalS12* do not have known physical positions, this work warrants suggestions for future studies. Regions with eQTL can be considered as targets for other studies searching for regions where the *CscalS* genes can be located. Equally important, there is the possibility of identifying other genes that are related to *CscalS* functions. The identification of hot spots reinforces the idea that the eQTL detected in this study may be influencing the expression of *CsCalS*. Additionally, any gene physically located in a hotspot is a candidate, possibly explaining the studied process.

The gene expression and eQTL mapping results revealed that reprogramming occurs in callose synthesis in *P. trifoliata* as well as in *C. sunki*. However, there is evidence that *P. trifoliata* does not accumulate or accumulates much less callose than *C. sunki* (Boava *et al*., 2017). Thus, we believe that *P. trifoliata* has mechanisms that prevent callose deposition.

## Conclusion

Despite the importance of eQTL mapping to provide a better understanding of the phenotypic variation (including those occurring during HLB), few related works exist in the literature. This study is the first to detect genomic regions associated with *CscalS* expression in plants infected with the causal agent of HLB disease.

The expression of all callose synthase genes was affected after *C*Las infection in the hybrid population studied. Thus, eQTL for *CscalS2, CscalS7, CscalS8, CscalS9, CscalS10*, and *CscalS12* were mapped in the *C. sunki* map and eQTL for *CscalS2, CscalS5, CscalS7, CscalS8, CscalS9* and *CscalS12* were mapped in the *P. trifoliata* map. eQTL analysis indicated that multiple regions can contribute to *CscalS* expression regulation and some eQTL have an epistatic effect for more than one *CscalS* gene. An important region was also verified on linkage group 8 that could influence the expression of *CscalS7* in plants affected by HLB.

The identification of hot spots reinforces the idea that eQTL identified in this study may influence the expression of *CscalS*. Additionally, any gene physically located in a hotspot is a candidate that can explain the studied process. This work suggests eQTL for *CscalS* related to HLB.

## Supplementary material

The following online material is available for this article:

Table S1 - Sequences of primer pairs used for RT-qPCR analysis.

Table S2 - Adjusted values of the expression of *CsCalS* 2, 5, 7, 8, 9, 10, 11 and 12.
