# Supplementary material for: Expression Quantitative Trait Loci (eQTL) mapping for callose synthases in intergeneric hybrids of Citrus challenged with the bacteria Candidatus Liberibacter asiaticus
Source: Genet Mol Biol. 2020 Jun 15;43(2):e20190133. doi: 10.1590/1678-4685-GMB-2019-0133 (PMC7295156; doi:10.1590/1678-4685-GMB-2019-0133)
Supplement: Table S1 [file 1415-4757-GMB-43-2-e20190133-s1.pdf]

Supplementary Material to "Expression Quantitative Trait Loci (eQTL) mapping for callose synthases in intergeneric hybrids of *Citrus* challenged with the bacteria *Candidatus* Liberibacter asiaticus"

Table S1 - Sequences of primer pairs used for RT-qPCR analysis.

Gene	Localization	Primer sequences (5’- 3’)	Reference
CscalS2	LOC102624514	F, ATCTCTGCCGGTTCTATGCG<br>R, CGGGCATCACTCTTTGACCT	Granato et al,. 2019
CscalS5	LOC102618167	F, GTGTGATTGAAACGGAAGCCA<br>R, CCATCATCACGCATAGGCCA	Granato et al., 2019
CscalS7	LOC102612996	F, GACGCCTAACCGAGTACCTGC<br>R, GTGCAGCTGGTGATCCATCA	Granato et al., 2019
CscalS8	LOC102631245	F, AGGATGTTTTCGCCGGTACA<br>R, ATCACGACCTTTGCCCACTT	Granato et al., 2019
CscalS9	LOC102612131	F, TCCTTTCTCGAATTGGCCGT<br>R, TGTCTGTGCGCGATATGAGG	Granato et al., 2019
CscalS10	LOC102616583	F, GGCTCGACTTGGCATACCTG<br>R, AACTGTTCCAAGCAAGGCGT	Granato et al., 2019
CscalS11	LOC102627313	F, GATGTGTACCGCTTGGGTCA<br>R, AGCAAGATAAAGACGCCCCC	Granato et al., 2019
CscalS12	LOC102610237	F, CTTGGGTCAGCGTGTTTTGG<br>R, CTCCTCGCAGTGTGCAGTTA	Granato et al., 2019
GAPDH	At1g13440	F,GGAAGGTCAAGATCGGAATCAA<br>R, CGTCCCTCTGCAAGATGACTCT	Mafra et al., 2012
FBOX	At5g15710	F, GGCTGAGAGGTTCGAGTGTT<br>R, GGCTGTTGCATGACTGAAGA	Mafra et al., 2012
