# Supplementary material for: Expression Quantitative Trait Loci (eQTL) mapping for callose synthases in intergeneric hybrids of Citrus challenged with the bacteria Candidatus Liberibacter asiaticus
Source: Genet Mol Biol. 2020 Jun 15;43(2):e20190133. doi: 10.1590/1678-4685-GMB-2019-0133 (PMC7295156; doi:10.1590/1678-4685-GMB-2019-0133)
Supplement: Table S2 [file 1415-4757-GMB-43-2-e20190133-s2.pdf]

Supplementary Material to "Expression Quantitative Trait Loci (eQTL) mapping for callose synthases in intergeneric hybrids of *Citrus* challenged with the bacteria *Candidatus* Liberibacter asiaticus"

Table S2 - The adjusted values of the expression for callose synthases 2, 5, 7, 8, 9, 10, 11 and 12.

Genotypes	Genes							
	CsCals2	*CsCals5*	*CsCals7*	*CsCals8*	*CsCals9*	*CsCals10*	*CsCal11*	*CsCals12*
1	1.95	1.99	1.10	0.56	1.38	1.08	1.24	0.09
10	0.32	5.19	1.15	#N/D	2.16	-0.19	1.27	0.33
101	0.82	-0.74	1.34	#N/D	1.18	0.04	1.22	-0.22
102	0.90	0.87	1.31	0.61	0.93	0.33	1.19	0.20
105	1.30	2.67	2.63	0.64	1.13	1.42	1.22	0.40
106	2.28	0.28	1.00	0.03	1.40	-0.16	1.22	0.16
107	1.22	-0.15	0.94	-0.74	0.69	0.94	1.21	-0.25
109	1.22	0.40	1.04	-0.62	1.05	1.14	1.22	-0.37
110	6.39	8.98	1.31	4.30	1.90	1.96	1.37	1.45
111	0.78	0.45	0.93	0.38	0.77	0.15	1.20	0.26
113	2.24	20.18	1.09	4.35	0.94	1.03	1.22	0.11
117	-0.56	0.50	0.96	-0.11	1.15	-0.16	1.22	0.07
118	1.38	1.06	1.01	-0.38	0.80	1.10	1.27	-0.27
119	1.98	0.11	1.06	-0.46	0.74	1.13	1.35	-0.10
121	1.02	0.38	0.73	0.02	0.35	1.56	1.20	0.23
124	3.32	0.58	1.55	0.32	1.46	0.20	1.24	0.90
125	3.21	1.56	2.74	10.43	2.53	1.65	1.52	3.93
126	-0.84	0.30	1.40	12.68	1.26	-0.32	1.32	0.39
129	3.20	-0.47	1.48	2.58	2.28	1.05	1.22	0.52
130	3.44	2.94	1.33	2.28	3.17	4.10	1.49	5.00
132	1.50	2.68	2.40	1.46	1.73	2.51	1.22	3.61
134	1.49	0.15	1.10	-0.63	0.66	1.00	1.22	-0.35
136	2.00	0.02	1.02	2.93	0.87	1.03	1.26	-0.07
137	1.90	0.56	1.10	0.37	1.13	2.26	1.25	1.54
14	-0.74	1.89	0.98	0.52	1.29	-0.31	1.21	0.13
141	8.00	3.54	1.59	2.23	3.69	1.58	1.22	3.50
142	3.47	3.08	1.18	1.62	1.13	1.33	1.33	0.21
143	18.08	9.77	3.10	0.74	2.07	54.37	1.34	4.86
146	7.34	0.56	1.57	0.34	4.94	1.98	1.33	1.29
148	1.33	-0.03	1.04	-0.41	1.25	1.09	1.21	0.39
149	2.29	-0.24	1.14	-0.23	0.90	1.04	1.32	0.34
150	-0.78	0.18	0.88	-0.23	1.08	-0.35	1.20	0.08
151	0.91	0.55	1.34	1.48	0.94	0.38	1.23	0.37

Genotypes	Genes							
	CsCals2	***CsCals5***	***CsCals7***	***CsCals8***	***CsCals9***	***CsCals10***	***CsCal11***	***CsCals12***
154	1.60	0.22	1.75	0.30	1.47	0.34	1.21	0.48
16	1.56	14.60	1.44	1.04	1.30	1.02	1.34	-0.37
163	2.05	0.69	1.72	0.61	1.24	1.19	1.32	1.22
173	1.64	1.10	1.45	5.68	1.53	0.91	1.20	-0.12
179	0.89	0.53	2.13	0.50	1.21	0.80	1.23	0.71
183	0.35	0.59	1.35	1.78	1.10	0.03	1.31	0.25
184	1.83	1.51	1.86	0.01	1.26	2.09	1.30	0.35
187	2.14	0.79	1.00	-0.41	1.22	1.39	1.22	0.37
189	1.29	0.41	1.70	0.68	0.48	0.24	1.26	0.36
19	6.92	-0.11	0.94	-0.06	3.23	0.97	1.21	-0.30
191	0.74	0.63	1.21	1.24	0.45	0.83	1.29	0.80
2	-0.09	0.89	1.06	0.11	1.44	0.49	1.24	0.13
20	-0.19	1.03	0.97	1.90	1.62	-0.24	1.29	0.16
217	0.91	0.19	0.89	0.22	0.50	1.33	1.20	0.40
23	0.95	0.04	1.29	0.80	0.84	0.06	1.20	-0.25
24	1.41	-0.06	1.46	7.91	1.75	1.01	1.20	0.54
26	1.18	1.11	1.89	0.38	1.22	0.21	1.25	0.40
279	1.22	0.42	0.79	0.85	0.60	2.28	1.21	0.46
28	1.88	4.71	0.77	#N/D	1.74	1.55	1.19	0.16
293	4.97	0.94	1.17	0.88	0.92	2.42	1.33	1.00
31	3.39	0.47	1.42	0.77	3.80	4.55	1.36	2.28
35	0.83	6.42	1.19	1.45	3.14	-0.28	1.25	0.37
4	2.52	0.75	1.67	#N/D	4.46	3.50	1.33	0.28
42	-0.66	0.31	0.92	1.15	1.85	-0.38	1.21	0.24
47	-0.51	2.02	0.84	0.16	1.76	-0.35	1.21	0.20
49	9.05	1.30	2.95	19.66	6.01	8.51	1.71	1.53
54	0.64	0.83	0.71	0.75	0.59	0.42	1.19	0.26
56	1.02	0.92	0.95	3.91	0.90	0.10	1.20	0.34
61	0.56	-0.01	1.30	1.80	1.09	0.43	1.40	0.59
66	0.47	-0.64	1.29	#N/D	1.04	-0.14	1.17	-0.26
68	3.64	-0.21	1.31	6.37	1.31	2.12	1.24	1.10
70	0.63	0.50	0.71	0.04	0.24	1.01	1.19	0.11
73	3.06	0.41	1.42	3.83	1.21	0.52	1.24	0.18
78	2.94	1.32	4.81	13.67	1.40	0.76	1.47	1.81
86	0.44	-0.78	1.29	#N/D	1.05	-0.15	1.18	-0.31
90	-0.19	0.81	1.16	2.60	1.24	-0.08	1.30	0.20
94	1.69	0.83	1.04	15.20	1.38	-0.06	1.28	0.33
96	-0.32	0.70	0.84	0.12	1.15	-0.15	1.20	0.11
99	3.78	0.60	1.37	0.86	0.94	0.33	1.30	-0.06
C. Sunki	2.79	0.34	2.11	0.80	1.66	1.94	1.39	1.33
P. trif	1.67	0.43	1.80	0.43	0.95	0.70	1.46	0.30
